# Resveratrol Induces Differentiation of Human Umbilical Cord Mesenchymal Stem Cells into Neuron-Like Cells

**DOI:** 10.1155/2017/1651325

**Published:** 2017-04-20

**Authors:** Li Guo, Liang Wang, Li Wang, Shi Yun-peng, Jing-jing Zhou, Zongmao Zhao, De-Pei Li

**Affiliations:** ^1^Department of Neurosurgery, The Second Hospital of Hebei Medical University, Shijiazhuang, Hebei 050000, China; ^2^Department of Pharmacy, The Fourth Hospital of Hebei Medical University, Shijiazhuang, Hebei 050011, China; ^3^North China University of Science and Technology Affiliated Hospital, Tangshan, Hebei 63000, China; ^4^Department of Anesthesiology and Critical Care, The University of Texas MD Anderson Cancer Center, Houston, TX 77030, USA

## Abstract

*Objective*. Human umbilical cord mesenchymal stem cells (hUC-MSCs) potentially differentiate to various types of cells including neuron-like cells. The natural polyphenol resveratrol benefits patients with many diseases including ischemic brain injury. We hypothesize that resveratrol induces differentiation of hUC-MSCs into neuron-like cells.* Methods*. Flow cytometry was used to determine the surface antigens in different stage of hUC-MSCs (P2, P5, and P10). Nestin, neuron-specific enolase (NSE), and glial fibrillary acidic protein (GFAP) were detected by immunocytochemistry, Western blotting, and real time RT-PCT. The cultured hUC-MSCs were treated with resveratrol at different concentrations (0, 7.5, 15.0, and 30.0 mg/L). Nestin, GFAP, and NSE protein and mRNA were measured at posttreatment time points of 2 h, 4 h, 6 h, 12 h, and 24 h.* Results*. Neuron-like cells were found in hUC-MSCs treated by resveratrol at concentrations of 15.0 and 30.0 mg/L, but not in hUC-MSCs treated with vehicle and 7.5 mg/L resveratrol. Furthermore, immunocytochemical staining revealed that nestin and NSE immunoreactivities were positive in resveratrol-treated hUC-MSCs at concentrations of 15.0 and 30.0 mg/L. Resveratrol treatment significantly increased nestin and NSE protein and mRNA levels 4 h after the treatment. However, resveratrol treatment did not change GFAP immunoreactivities and protein and mRNA expression levels in cultured hUC-MSCs.* Conclusions*. Taken together, resveratrol treatment induces a differentiation of hUC-MSCs into neuron-like cells at relatively high concentrations.

## 1. Introduction

The mesenchymal stem cells (MSCs), derived from the mesoderm in early development, are pluripotent stem cells that have the properties of multiline age differentiation [1, 2]. These MSCs are able to differentiate into bone cells, adipose cells, skeletal muscle cells, cardiac muscle cells, nerve cells, and epithelial cells [1, 2]. MSCs can be found in many tissues including skin, fat, muscle, placenta, amniotic fluid, umbilical vein endothelial under layer, liver, and blood [3]. Although MSCs are enriched in bone marrow, the practical source of obtaining MSCs is fetus umbilical cord blood [4]. Recent studies have shown that the human umbilical cord mesenchymal stem cells (hUC-MSCs) have many advantages including excellent potential of differentiation, low immunogenicity, convenient transfection, rich source, and less trauma, no ethical constraints, and relatively easier accessibility, when they are compared with the MSCs from other sources. Thus, hUC-MSCs have great potentials for clinical application.

The most commonly used approaches to induce differentiation of MSCs into neuron-like cell include (1) antioxidant agents such as mercaptoethanol, dimethylsulfoxide (DMSO), and beta hydroxy acid (BHA); (2) neurotropic factors such as retinoic acid (RA), nerve growth factor (NGF), epidermal growth factor (EGF), basic fibroblast growth factor (bFGF), brain derived neurotropic factor (BDNF); (3) Chinese herbs and their effective components such as astragalus glycosides, berberine, tanshinone, lycium barbarum polysaccharide, acanthopanax; (4) cultural method; and (5) gene transfection: transfection of specific genes such as Noggin [5] and Notch into hUC-MSCs to get neuron-like cell. However, these differentiation approaches are of low efficiency.

Resveratrol (3,5,4′-trihydroxy stilbene) is one of the polyphenols extracted from grape, polygonum cuspidatum, semen cassia, or peanut. It has been used clinically to treat cardiovascular disease, slow down cancer process, reduce ischemic brain injury, enhance antibiotic action, and exert estrogenic effects [6–9]. Furthermore, resveratrol modulates immune responses, reduces allergic reaction, delays aging process, and dispels chloasma [10, 11]. In addition, resveratrol has relatively strong antioxidative effect through removing free radicals [12]. Resveratrol can increase the nerve protective effect [13]. On the basis of previous studies, we tested our hypothesis that resveratrol induces differentiation of hUC-MSCs into neuron-like cells. In this study, we also determine the optimized concentration for resveratrol induced differentiation of hUC-MSCs into neuron-like cells.

## 2. Materials and Methods

### 2.1. Materials

Resveratrol (purity: 99.2%) was purchased from Nanjing Zelang Pharmaceutical Co., Ltd. (Nanjing, China). Dulbecco's Modified Eagle's Medium (L-DMEM)/F-12 and fetal bovine serum (FBS) were purchased from Gibco (Grand Island, NY, USA) and trypsin was obtained from Amresco LLC (Solon, OH, USA). Fluorescein isothiocyanate (FITC)-CD19, FITC-CD34, phycoerythrin- (PE-) CD11b, PE-CD73, PE-CD90, PE-CD45, PE-CD105, and glial fibrillary acidic protein (GFAP) were obtained from BD Biosciences (Franklin Lakes, NJ, USA). Nestin and neuron-specific enolase (NSE) were purchased from Cell Signaling Technology Inc. (Danvers, MA, USA). Anti-rabbit immunoglobulin G (IgG) (1 : 2,000) was purchased from Affinity Biosciences (Cincinnati, OH, USA). PS immunohistochemistry kit was purchased from Beijing Zhongshan Golden Bridge Biotechnology Co. Ltd. (Beijing, China). Taq polymerase chain reaction (PCR) star mix was purchased from Beijing GenStar Biosolutions Co. Ltd. (Beijing, China); EasyScript First-Strand cDNA synthesis supermix was obtained from TransGen Biotech Co., Ltd. (Beijing, China).

### 2.2. Isolation and Culture of hUC-MSCs

Umbilical cord was rinsed with D-Hank's medium thoroughly and then stored in H-DMEM/F-12 culture medium under aseptic conditions at 4°C. The blood vessel including artery and vein were removed. The umbilical cord mesenchymal tissue was cut into small pieces in the size of 1 mm and was digested by using 0.2% collagenase II. To obtain the primary cells, the digested tissue in the solution was placed in a culture flask containing 2.0 ng/ml EGF, 20% FBS, 25.0 mM L-Glu, and 100.0 U/ml penicillin-streptomycin mixture at 37°C with 5% CO_2_ and saturation humidity. The culture medium was then half-changed every 24 hour and replenished every 3 days. When 80–90% confluence was achieved, cells were then rinsed twice with phosphate-buffered saline (PBS) containing trypsin (0.25%) and EDTA (0.2 g/l) to further digest into single cells for passaging at the ratio of 1 : 3. The culture medium of H-DMEM/F-12 contained 100 U/ml of a penicillin-streptomycin mixture and 10% FBS [14].

### 2.3. Analysis of Cellular Phenotype of hUC-MSCs

In the logarithmic phase of hUC-MSCs growth, trypsin was used to digest the hUC-MCCs to individual cells, and the suspension was aliquot into tubes containing 1 × 10^6^ cells/tube. The mouse anti-human monoclonal antibodies CD11-PE, CD105-PE, CD73-PE, CD45-PE, CD90-PE, CD19-FITC, human leukocyte antigen- (HLA-) DR-PE, and CD34-FITC (each 5 *μ*l) were, respectively, added to 8 tubes. Anti-mouse IgG1-FITC and anti-mouse IgG1-PE (each 7 *μ*l) were applied in other 2 tubes as isotype controls. These tubes were then well mixed and incubated for 30 min at 4°C. The isolated cells were then analyzed by flow cytometry.

### 2.4. Differentiation of hUC-MSCs into Neuron-Like Cells

At the P5 stage, the hUC-MSCs were passed into six-well plates and 25 cm^2^ cell-culture bottle with the complete culture medium. One six-well plate and three 25 cm^2^ culture bottles were selected randomly into one group. Four groups of hUC-MSCs were treated with resveratrol at concentrations of 0.0, 7.5, 15.0, and 30.0 mg/L in L-DMEM culture medium. The expressions of GFAP, nestin, and NSE were detected by immunohistochemical staining at the 4 h treatment. The mRNA and protein expressions of GFAP, nestin, and NSE were determined by RT-PCR and Western blot at 2 h, 4 h, 6 h, 12 h, and 24 h after induction.

### 2.5. Identification of Differentiated Cells

The nestin, NSE, and GFAP were detected and analysis by immunocytochemistry after 4-hour incubation. Briefly, the cultured cells were gently rinsed once with PBS and then fixed with 4% paraformaldehyde at room temperature for 20 min. After rinsing three times with PBS, the cells were immersed into 0.5% Triton X-100 in PBS for 15 min followed by incubation with 3% H_2_O_2_ at room temperature for 5 min. The cells were blocked with normal goat serum blocking agent for 15 min and then incubated with primary antibodies against NSE, GFAP, and nestin at 4°C overnight. The appropriate biotinylated secondary antibodies were then applied for 15 min and then incubated with horseradish peroxidase-conjugated streptavidin for 1 min. Finally, the cells were stained with freshly prepared DAB for 1 min and counterstained with hematoxylin.

### 2.6. Western Blot Analysis

Cell lysates were obtained by using RIPA buffer and phenylmethylsulfonyl fluoride (Beijing Solarbio Science & Technology Co., Ltd.). The protein concentrations were quantified by an ultramicro ultraviolet visible light meter (Gene Company, Ltd.). Each sample of 16 *µ*g protein was loaded and electrophoresed in 10% SDS-PAGE gels, which were transferred to polyvinylidene fluoride membrane (PVDF) (Beijing Solarbio Science & Technology Co., Ltd.). After being blocked with 10% nonfat milk for 2 hours, the PVDF membranes were incubated with primary antibodies against GFAP, nestin, and NSE at 4°C overnight. On the second day, the membranes were incubated with anti-rabbit IgG (1 : 2,000; Affinity Biosciences). Images were visualized by SuperSignal West Pico Chemiluminescent Substrate (Thermo Fisher Scientific, Waltham, MA, USA) and captured by using the Image AlphaEaseFC system (Alpha Innotech Co., San Leandro, CA, USA).

### 2.7. RT-PCR

Total mRNA was extracted by TriPure Reagent (Aidlab Biotechnologies Co., Ltd., Beijing, China) and quantified by ultramicro ultraviolet visible light meter (Gene Company, Ltd., Hong Kong, China). Total mRNA were first reversely transcripted to cDNA by using EasyScript First-Strand cDNA synthesis supermix (TransGen Biotech Co., Ltd.). Then the following protocol was used for semiquantitative PCR: 1 cycle of 94°C for 3 min; 30 cycles of 94°C for 30 sec, and 72°C for 30 sec; and a final extension at 72°C for 10 min. The PCR product was separated by electrophoresis, and the interested genes were normalized by GAPDH transcription. The primers were designed by Primer 5.0 as the below: nestin Forward: TCCAGAAACTCAAGCACCACT Reverse: TCCACCGTATCTTCCCACCT 342 bp; NSE Forward: GGCACTCTACCAGGACTTTG Reverse: GCGATGACTCACCATAACCC 286 bp; GFAP Forward: GTCCATGTGGAGCTTGAC Reverse: CATTGAGCAGGTCCTGGTAC 406 bp; GAPDH Forward: AGAAGGCTGGGGCTCATTTG Reverse: AGGGGCCATCCACAGTCTTC 258 bp.

### 2.8. Statistical Analysis

All the data were expressed as Mean ± SEM. Statistical analysis was performed by SPSS 21.0. One-way ANOVA was performed in three or more groups comparison followed by Student-Newman-Keuls method (*q*-test) between groups. If the data distribution did not fit the normality or homogeneity of variance, the nonparametric rank sum test (K-W *H* test) was the performed. *P* < 0.05 was considered to be significant difference.

## 3. Result

### 3.1. Resveratrol Induced Morphological Changes of hUC-MSCs into Neuron-Like Cells

**Figure 1:**
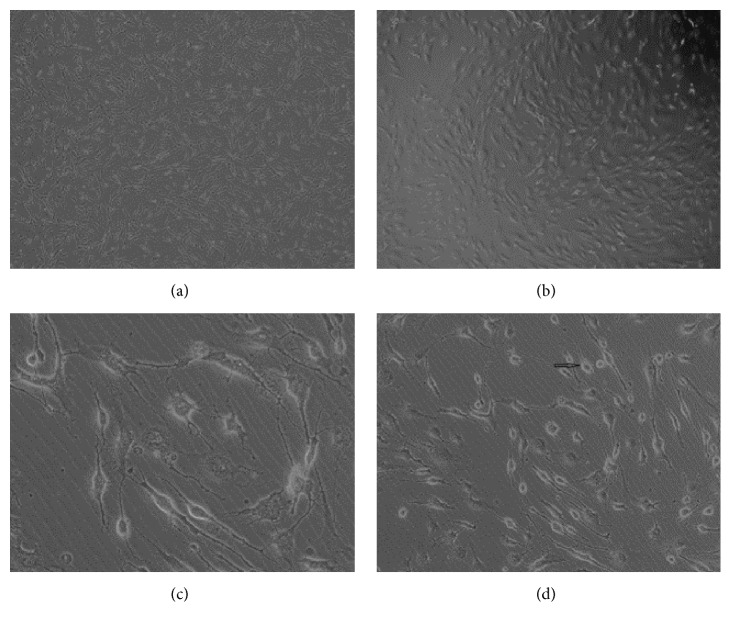
Images showing that different concentrations of resveratrol (0.0 mg/L (a), 7.5 ml/L (b), 15.0 mg/L (c), and 30.0 mg/L (d)) induce differentiation of hUC-MSCs into neuron-like cells.

Primary cultured cells were passaged at a ratio of 1 : 1 and the hUC-MSCs were passaged at the ratio of 1 : 3 or 1 : 2. After being passaged continuously, the hUC-MSCs still had strong ability of proliferation. There was no significant change in hUC-MSCs treated with resveratrol at 7.5 mg/L compared with the control condition. Treatment with resveratrol (15.0 mg/L) for 6 h changed the shape of hUC-MSCs from circular to bipolar cells in oval or quasi-circular. The ratio of neuron-like cells was about 5% (Figure 1). However, with resveratrol (30.0 mg/L) treatment of hUC-MSCs for 1 h, the ratio of neuron-like cells reached 50%. The number of multistage cells is significantly higher than the numbers in hUC-MSCs treated with resveratrol at 15.0 mg/L. Furthermore, the cells appeared connected into a net-like pattern (Figure 1). After 6 h treatment with resveratrol (30 mg/L), 85%~90% of the hUC-MSCs displayed neuron-like shape (Figure 1). However, the ratio of neuron-like cells reduced to 70% and 80% after 24 h treatment with resveratrol.

### 3.2. Cell Surface Markers

**Figure 2:**
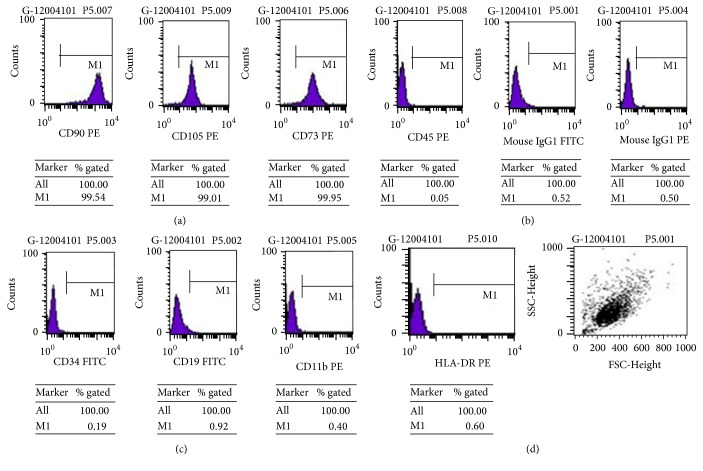
Fluorescence-labeled cell sorting analysis from hUC-MSCs of (a) CD90, CD105, and CD73; (b) CD34, CD19, and CD11b; (c) CD45; and HLA-DR and FSC-Height.

We analyzed the surface antigens at the stages of P2, P5, and P10 of hUC-MSCs with flow cytometry. CD105, CD73, and CD90 were expressed on hUC-MSCs at the stages of P2, P5, and P10. Stages P7 and P8 showed signs of reduced ability of proliferation compared with P2 to P5. Thus, we choose to use P5 to represent the early passages to test the effect of resveratrol on the cell surface markers. In addition, no apparent morphological changes of hUC-MSCs were observed from stages of P5 to P8. CD11b, CD45, CD34, CD19, and HLA-DR were negative in hUC-MSCs at stages of P2, P5, and P10 (Figure 2).

### 3.3. Resveratrol Induced Immunoreactivities of Nestin, NSE, and GFAP

**Figure 3:**
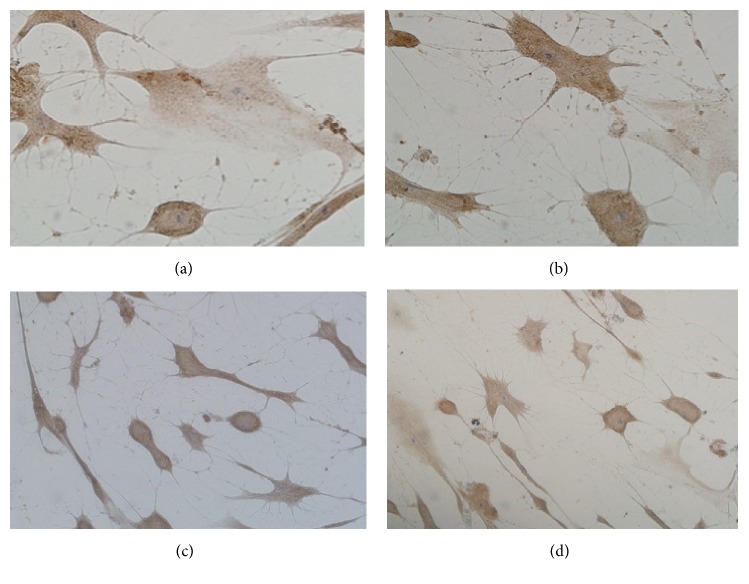
Immunostaining images showing the effect of resveratrol (15.0 and 30.0 mg/L) on neuron markers nestin and NSE on hUC-MSCs. (a) Nestin and (b) NSE immunochemical staining of hUC-MSCs treated with resveratrol at a concentration of 15.0 mg/L. (c) Nestin and (d) NSE immunochemical staining of hUC-MSCs treated with 30.0 mg/L resveratrol.

Nestin and NSE are specifically expressed on neurons and have been used as putative neuronal markers; we determine the effect of resveratrol on immunoreactivities of nestin and NSE in cultured hUC-MSCs. The hUC-MSCs were immunostained with antibodies against nestin and NSE. The brown color cell bodies and purple nucleus were nestin- or NSE-positive cells (Figure 3). Nestin or NSE-positive immunoreactivities were found in hUC-MSCs treated with resveratrol at a concentration of 15.0 and 30 mg/L. To determine if resveratrol differentiates hUC-MSCs into glia, GFAP immunoreactivity was also determined by using antibodies against GFAP. GFAP immunoreactivities were negative in hUC-MSCs treated with vehicle and resveratrol at all concentrations (Figure 3). Resveratrol treatment increased the nestin and NSE-positive cell number at concentrations of 15.0 and 30.0 mg/L compared with the vehicle-treated cells (*P* < 0.01). However, 7.5 mg/L resveratrol treatment did not change nestin and NSE-positive cell numbers (*P* > 0.05).

### 3.4. Resveratrol Increased mRNA and Protein Levels of Nestin and NSE

**Figure 4:**
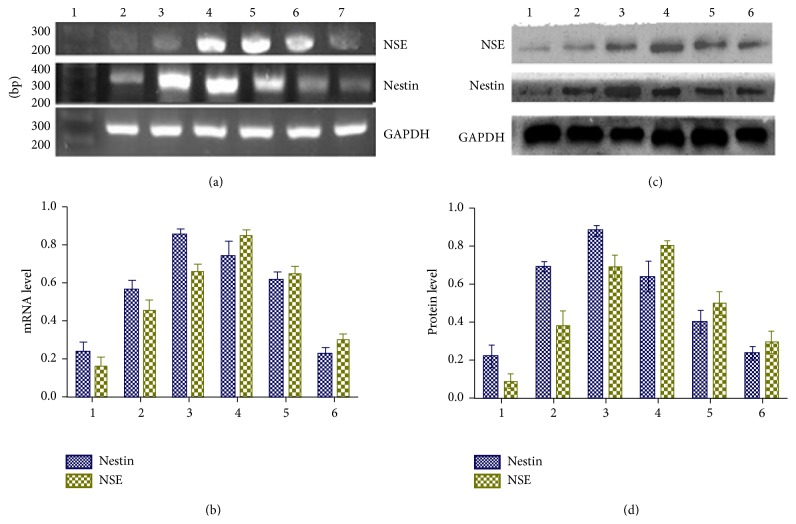
mRNA and protein levels of nestin and NSE in resveratrol-treated hUC-MSCs. (a) Original gel images and (b) summary data showing mRNA levels of nestin and NSE in resveratrol-treated hUC-MSCs at different time points. (c) Gel images and (d) summary data showing protein levels of nestin and NSE in resveratrol-treated hUC-MSCs at different time points. 1–6: period of resveratrol treatment for 0 (control), 2 h (3), 4 h (4), 6 h (5), 12 h (6), and 24 h (7).

We then measured mRNA level of GFAP, nestin, and NSE in vehicle-treated and resveratrol-treated cultured hUC-MSCs. The GFAP, nestin, and NSE mRNA levels were measured by RT-PCR before and after treatment with resveratrol (30 mg/L) for 2 h, 4 h, 6 h, 12 h, and 24 h. The GFAP mRNA was not detectable in hUC-MSCs treated with vehicle and resveratrol at all concentrations. Nestin mRNA levels were gradually increased and reached a peak value at 4 h after resveratrol treatment. Furthermore, NSE mRNA levels were also increased and reached the peak value when hUC-MSCs were treated for 6 h (Figure 4).

Consistently with these changes of nestin or NSE mRNA levels in response to treatment with resveratrol, the protein levels of nestin or NSE have similar changes in response to resveratrol treatment. Resveratrol treatment increased the protein levels of nestin and reached a peak value 4 h after resveratrol treatment. Also, resveratrol treatment increased NSE protein levels and reached a peak value 6 h after resveratrol treatment (Figure 4).

## 4. Discussion

Many agents induce differentiation of MSCs to neuron-like cells through distinct mechanisms. For example, *β*-mercaptoethanol induces MSCs to differentiate into neuron-like cells through modulating cAMP concentration to increase protein kinase A (PKA) activity [15]. It has been shown that EGF directly induces differentiation of MSCs and promotes mitosis to maintain growth and survival of synapses [16]. The acanthopanax suppresses p65 trafficking from the cytoplasm towards the cell nucleus to inhibit the downregulation of I*κ*B*α*. Also, acanthopanax inhibits NF-*κ*B to relieve the obstruction of differentiation of stem cells to promote differentiation [17, 18]. Chen et al. [19] have demonstrated that the activation of gene GAP-43 importantly regulates differentiation through influencing the growth and extension of synapses, from which the injured nerve starts to repair. Wu et al. [20] have confirmed that miRNA-128 induces differentiation of BMSCs into neuron-like cells when Wnt3a gene expression is downregulated.

Resveratrol exerts many pharmacological functions such as anticardiovascular diseases, antitumor, anti-inflammation, neuron-protection, antiaging, and antioxidation. Previous studies have shown that resveratrol affects the expression levels of proangiogenic related factor in the recovery phase of ischemia-reperfusion brain tissues and exerts positive effect on hemodynamics and revascularization in ischemic brain tissue [21]. Furthermore, resveratrol inhibits oxidative stress via activation of SIRT1 [22–24]. It has been shown that resveratrol may decrease the expression levels of MMP-2 through inhibiting the activity of NF-*κ*B and subsequently reducing the invasion of glioma [25, 26]. In addition, resveratrol reduces the number of macrophages and neutrophils, inhibits H-thymidine, and induces tumor cells to differentiate into nonpropagative phenotype cells. These studies suggest that resveratrol inhibits development of tumor [27]. Resveratrol at different doses can alleviate the injury in rat cortical neural stem cells, which is caused by oxygen glucose deprivation/reoxygenation (OGD/R) in vitro in different extent. In addition, resveratrol can promote the proliferation of cortical neural stem cells [28].

This is the first study showing that resveratrol treatment induced differentiation of hUC-MSCs into neuron-like cells. In this study, we observed the cellular markers CD 105, CD73, and CD90 rather than other cellular and molecular traits for cell senescence. These markers did not show any changes in response to resveratrol treatment compared with vehicle treatment. However, we cannot rule out the possibility that other cellular and molecular traits are altered in response to resveratrol treatment. It is very important to consider if hUC-MSCs at stage of P5 exhibit several signs of senescence in the future studies. In this study, we detected the corresponding neural markers nestin and NSE by observation of related-positive immunohistochemistry staining and the expression levels of their mRNA and protein. The negative expression of GFAP in hUC-MSCs indicates that resveratrol did not induce differentiation of hUC-MSCs into glia cells. We also determined the optimal treatment time and concentrations for resveratrol to induce hUC-MSCs differentiating into neuron-like cells. We found that resveratrol treatment significantly increased the mRNA and protein expression levels of neuronal markers nestin and NSE. Furthermore, the nestin mRNA and protein expression levels reached peak values after treatment with resveratrol for 4 h, while the NSE mRNA and protein expression levels reached peak values after treatment with resveratrol for 6 h. In addition, we found that the expression of GFAP was negative in both resveratrol-treated and vehicle-treated hUC-MSCs, indicating that resveratrol treatment did not induce hUC-MSCs differentiation into glia cells. Although previous studies have shown that hUC-MSCs induce themselves to express their associated neural markers partially [29], we found very low expression levels of neuronal markers nestin and NSE in vehicle-treated and low resveratrol-treated hUC-MSCs in our preparation.

In this study, we found that a suitable concentration for resveratrol (30 mg/L) induced hUC-MSCs differentiation into neuron-like cells. Although resveratrol activates signaling pathways involving cAMP, Epac1, and AMPK to activate SIRT1 indirectly, it is well accepted that the beneficial effect of resveratrol is through directly activating SIRT1 [30–32]. It is not clear which signaling pathways are involved in resveratrol induced differentiation of hUC-MSCs into neuron-like cells. Thus, further studies are warranted to determine these mechanisms.

In summary, findings from our study suggest that the resveratrol can induce hUC-MSCs to differentiate into neuron-like cells effectively at certain concentrations. This novel finding provides detailed information on resveratrol induced hUC-MSCs differentiation into neuron-like cells. This information may help to develop stem cell implantation-based therapy for ischemic neuronal damage.
